# Committee experiences of using formal consensus in healthcare guidelines: a longitudinal qualitative study

**DOI:** 10.1186/s12911-023-02220-5

**Published:** 2023-08-02

**Authors:** V Roberts, Patrice Carter, P Barnett, MA Mugglestone, S Pilling

**Affiliations:** 1 Centre for Outcomes Research and Effectiveness, Research Department of Clinical, Educational & Health Psychology, University College London, 1-19 Torrington Place, London, WC1E 7HB UK; 2 National Guideline Alliance, Royal College of Obstetricians and Gynaecologists, 10-18 Union Street, London, SE1 1SZ UK

**Keywords:** Formal consensus methods, Group decisions, Guidelines, Qualitative research

## Abstract

**Background:**

This feasibility study has the primary aim of capturing and comparing participant expectations and experiences of using a formal consensus method (FCM) and to explore whether these views change following participation within a guideline committee where FCM are used.

**Methods:**

Twelve healthcare committee members and associated technical team members participated in semi-structured qualitative interviews before and after using FCM during guideline committee meetings. Interviews also focused on past experiences and expectations of informal consensus methods.

**Results:**

Participants said formal consensus included a greater range of evidence. They described positive reactions and found it a useful way to encourage involvement by balancing group power dynamics. Group discussion time was identified as important to clarify ideas, supported by good group chairing. However, participants reported that undertaking FCM required additional resources and suggested targeting its use for low quality evidence, limited committee expertise, or where the evidence is controversial.

**Conclusions:**

FCM is an acceptable alternative to informal consensus methods that has qualities specifically helpful to healthcare guidelines such as encouraging participation, inclusivity of a broad range of evidence, and managing group dynamics. More research is required to better understand when using formal consensus is most appropriate and effective.

**Supplementary Information:**

The online version contains supplementary material available at 10.1186/s12911-023-02220-5.

## Background

Healthcare guidelines in the UK are generally created using the best available evidence (i.e., systematic literature reviews of published randomised controlled trials) as per the NICE manual [1]. In areas of weaker evidence (i.e., where there is limited trial evidence, or conflicting results) the incorporation of data from other sources such as committee member expertise becomes especially important, and the quality of this depends on the extent of contribution and engagement from individuals, [2] and individual biases in the interpretation of evidence [3–7]. In general, group decisions are based on informal consensus, which involves committee members freely interacting and non-structured discussion, with no instructions on how to reach consensus [8]. Formal consensus methods (FCM) have been developed to manage unstructured data from multiple sources including expertise and empirical data, which can be particularly important for the development of healthcare guidelines [9]. FCM aim to enhance group decision-making by increasing group member participation, task efficiency, and offering a transparent and systematic approach by separating solution generation and evaluation phases [8, 10, 11].

Commonly used FCMs in healthcare are the Nominal Group Technique (NGT), [12] Delphi, [13] and RAND [13]. In brief, NGT is a structured interactive process within a group, where participants record their opinions independently and privately without discussion, a facilitator collects the opinions and these are then discussed in a structured format, participants then privately record judgements, and the process is repeated; judgements on statements are aggregated to derive final group consensus [6, 8, 14]. In contrast to the NGT, participants taking part in Delphi do not meet in person, participants are sent clearly defined questions for self-completion. Responses are collated and then sent back to participants in summary form, indicating group judgement and their individual response, participants can then revise their response having reviewed group feedback; the process can be repeated several times until aggregated data demonstrates consensus [6, 8, 14]. The RAND version is a modified NGT, where participants are sent questionnaires to rate independently and privately, the participants are then brought together in person to discuss views, following which they again privately record their own views [8]. NGT may offer advantages of speed and complex problem solving when compared to Delphi, and flexibility of application when compared to RAND [15]. FCM have been suggested for guidelines that have limited evidence because they can provide a transparent way of combining evidence from multiple sources including expert opinion [6, 8, 16]. The current study was based on NGT methods designed for use in healthcare guidelines [6, 17, 18]. Although a major strength of FCM is assumed to be an increased participation of group members [19, 20] there has been no qualitative research investigating the perspectives of those using FCM in a healthcare guideline setting.

### Study aim

This feasibility study has the primary aim of capturing and comparing participant expectations and experiences of using FCM, and to explore whether these views change following participation within a guideline committee where FCM are used.

## Methods

This study was a longitudinal qualitative design, involving two time points with two separate groups that had planned to use FCM. Committee members in these groups were invited to take part in interviews before and after the FCM exercise. Participants were invited to take part in the research verbally during a committee meeting which was conducted prior to the committee meeting where the FCM was carried out. All committee members and technical team members then received an email invite which included study details; those interested in participating responded to the email and provided consent to take part in the study. All participants gave written consent. The study was approved by the University College London (UCL) ethics committee (Ref CEHP2018569).

### Participants

Participants were recruited based on purposive sampling; [21] all guideline committee members and technical team members working on the guidelines were considered suitable for invite to the study. Purposive sampling of this group was deemed appropriate due to individuals knowledge of the guideline development process. Twenty-one individuals consented to be interviewed, of these, twelve participated in the interviews prior to the FCM process, and data was deemed saturated as similarities in responses were seen. The same twelve participants were invited back for post-FCM interviews; however, only nine of these individuals also completed the post-FCM interviews. The two samples are described in the Appendix 1. Participants were grouped by their role in their committee. For example, the guideline committee were comprised of medical doctors (categorised as “doctors”), nurses, pharmacists, and dieticians (categorised as “other healthcare professionals” (OHP)) and lay-members. The technical team were comprised of health economists, systematic reviewers, and guideline leads.

### Interviews

Two researchers (VR, PB) conducted semi-structured interviews of 30–40 min. The topic guide was developed iteratively over the course of several pilot interviews with members of the research team. Following the development of the topic guide, interviews were audio-recorded and transcribed verbatim. The pre-FCM interviews focused on participant past experiences of formal and informal consensus methods, and their expectations of FCM overall and the NGT method. The post-FCM interviews focused on participant experiences of using FCM within the committee meeting, its effectiveness and relevance within the healthcare guideline setting. The FCM was conducted by NGA technical team members using methods described elsewhere; [17] interview questions are provided in the Appendix 2.

### Data analysis

The data were anonymised and uploaded to NVivo V. 12 (QSR International) for analysis. Transcripts were independently coded by two researchers (VR, PB), initially by coding for individual units of meaning before identifying higher-order groupings. These codes were developed in an iterative manner, with several rounds of coding and cross-examination between researchers. Inter-rater reliability checks for themes were completed on 50% of the transcripts and achieved a minimum of 85% agreement.

## Results

We created two main themes in order to aid understanding of discussion points; these are ‘Theme cluster 1: Consensus process’ which relate to the evidence generation and general methodology of conducting formal consensus. ‘Theme cluster 2: Group process’ incorporates discussion points relating to the group interaction required for formal consensus. Within each cluster we present participants results prior to the committee meeting (pre-FCM comments) and then after (post-FCM comments); demonstrating similarities and differences in opinions before and after the FCM.

### Theme cluster 1: consensus process

#### Pre-FCM comments

Balancing guideline requirements with the research evidence

Participants described the impact of gaps in the evidence on developing guidelines. For informal consensus methods, this was in the context of a lack of a structured approach to managing uncertainties in the data.

“I think there are people around who think you shouldn’t write a guideline unless there’s definite evidence… In many ways it’s the other way around. You need guidance when there isn’t a controlled trial because that’s why it’s harder to pin down” A3 Pre-FCM.

#### Post FCM comments

Statements can be repetitive or ambiguous but focus discussion

It was generally reported that the FCM statements helped focus the discussion and translate opinion into useable recommendations.

“Yeah, I think they were good, worked easily, then in the end to recommendations that were made.” A15 Post-FCM.

However, participants felt some statements were ambiguous or repetitive. Several interviewees questioned whether this was because published guidelines rather than systematic reviews were used as evidence sources.

“The evidence statements had come from other similar statements by other bodies. So, it’s kind of a consensus statement on the top of an existing public consensus. We finished up… with a set of bland statements, which are kind of just giving an importance to, what is generally accepted practice, and has been for a long time.” B5 Post-FCM.

#### Themes across pre and post FCM comments

Pre- and post-FCM comments shared themes regarding the effort and resource intensiveness of FCM, and the application of the method.

Effort and resource intensiveness

Pre and Post interviews discussed the resource intensiveness and difficulties with learning a new consensus method. However, they differed in that pre-FCM interviews referred mainly to committee lack of confidence and apprehension as a barrier to implementation, and post-FCM interviews focused on the increased time for implementing and learning a new process, particularly for technical staff.

“So, I feel, overall, it was worthwhile… because it’s quite a new process, I think the committee perhaps weren’t super-confident with it… I think it just needs to be used perhaps more often… when something’s kind of new and unfamiliar, there can be a bit of apprehension about it.” A18 Post-FCM.

“I’m nervous that the statements aren’t going to be good enough and accepted by the committee… I’m not a clinician. I don’t have the same expertise clinicians would have” A18 Pre-FCM.

Appropriate Application and Credibility

Interviewees identified FCM as a way of increasing credibility of decision-making by using a transparent and robust process. They also called for greater clarity of when to best apply FCM.

“…Do think that there’s a lot of suspicion about the way in which closed groups come to decisions… you can say, in the absence of clinical data this is the robust way in which we have come to a decision as a group; we haven’t just sat there and chit-chatted over tea and biscuits”. B16 Pre-FCM.

“I’m not against it, I think it’s good. It just needs to be more clearly defined and it needs to be sort of like- I still think it should be the exception rather than the rule”. A15 Pre-FCM.

Following the FCM experience, participants suggested that the application of FCM was dependent on committee expertise, group cohesiveness, whether there is limited or low-quality evidence, or when there is controversy about the evidence.

“The problem is when you have evidence and the evidence is conflicting and controversial and there are different views in the room, and then it’s when you need these methods to formalise what you want to say and what you want to recommend.“A7 Post-FCM.

“It would depend on the group… I think probably if you’ve got people who are a bit afraid to talk and things like that NGT might be a bit more helpful.” B11 Post-FCM.

Post-FCM interviews highlighted the importance of investigating FCM in a range of topics to better understand the appropriate application of FCM.

“It’s a little bit difficult to disentangle the method from, you know, the topics that were being discussed. Because they were fairly straightforward topics.” A10 Post-FCM.

### Theme cluster 2: group process

#### Pre-FCM comments

Anonymity benefits

Participants discussed anonymity as being important for managing power dynamics and increasing sharing of views.

“…was it anonymous, these comments? … Yes, so definitely that could help” A13 Pre-FCM.

Continuity of group members

Individuals spoke of the challenges of updating committee member absences on meetings.

“…it’s difficult to get the entire panel together for every meeting, so I do find that sometimes if a particular member has not been at the previous meeting and we may revisit the same topic at the subsequent meeting and they are there we end up going back round almost the same discussion”. A13 Pre-FCM.

### Post-FCM comments

The dangers of purported efficiency

Post-FCM interviews questioned the efficiency of FCM, noting the negative impact on reducing discussion time and risk of absent group members missing more of the guideline.

“I think they were worried that someone was missing from that day…So then I think she had to be emailed later on to be asked if she was okay with how the first round went.” B21, Post-FCM.

No difference between FCM and informal consensus methods

A final theme present in the post-FCM interviews was a lack of difference between FCM and non-FCM discussions. Participants said this was driven by factors other than the FCM method. The FCM question was broader than average which had an impact on the process in general.

“You’re asking me to compare what it’s like to have no data to making recommendations where you’ve got loads of data… I think it’s very different in terms of how robust the making recommendations would have been.” B20, Post-FCM.

The broader question made little difference to the FCM committee meeting but increased the technical team workload who prepared the FCM statements for the meeting.

“They’re similar in terms of how the committee would have handled what they were doing. I don’t think it’s similar in terms of what the technical team, would have had to have done, because obviously there’s a lot of work involved in us having to deal with that.” B20 Post-FCM.

Another factor participants said reduced comparability between conditions was that there was extensive agreement in the topic area of the FCM condition.

“I don’t think it made a huge amount of difference because there wasn’t a huge amount of disagreement… there was a consensus that these were all sensitive things, and they were important and couldn’t be left out.” B5 Post-FCM.

### Both pre- and post-FCM comments

Management of expertise/ Equal Participation

Participants in both pre- and post-FCM interviews identified that a formal approach improved participation.

“where there’s a less structured approach, some people will feel more confident about speaking out and putting forward their point of view than others…we’re all there because we’ve got a particular interest in this field …because somebody speaks, who’s very passionate about what they’re saying, and you think, ‘I’m not sure I can say anything, I can put another point of view because of that.’” A10 Pre-FCM.

“People can jot down what their views are without feeling that they’re going to be singled out by, oh your view is nonsense… because it basically comes back, this is the percentage; so you don’t know who the 10% are who didn’t agree with the statement.” A7 Post-FCM.

Chair leadership

Both pre- and post-FCM participants named the leadership from the chair as an essential component for effective group decision-making.

“…the chair… does not allow people to dominate, but some other times chairs are more gentle, so they will not stop people”. B22 Pre-FCM.

Discussion clarifies

People also identified discussion during group decision-making as essential to help clarify thinking.

“…I think discussion is always good because it’s dynamic and because you have that instant response to somebody else’s thoughts” A10 Pre-FCM.

“because there was a chance for discussion as well, it meant that we could clarify the question that was being asked or the statement that was being made. “ A10 Post-FCM.

However, time allocated to discussion required flexibility depending on the topic area, and a balance needed to be struck to allow the exchange of multiple perspectives alongside focused and structured discussion.

“I did find it frustrating because it was, well, okay this is what the consensus has been, and then the door was shut on further discussion if you felt strongly that it didn’t quite match what the data showed.” A10 Pre-FCM.

Positive Experiences

Positive experiences were a theme across both data sets. People described committee discussions as enjoyable with reference to learning a new method (pre-FCM interviews), or the utility of the FCM process (post-FCM interviews).

“….I happen to be one of the people that knows the evidence on most things reasonably, well, but it’s always quite interesting to hear people chip in from a different perspective and I think that’s a healthy thing.” A3 pre-FCM.

“I think in the end, we made recommendations, so I think that’s a good thing, and I think the process adds weight to recommendations, which otherwise wouldn’t have had evidence to support them. So I feel, overall, it was worthwhile.” A18 Post-FCM.

## Discussion

This study aimed to capture participant expectations and experiences of using FCM during guideline development. Overall, pre- and post-FCM themes were consistent, and in-line with previous research on the use of formal consensus methods across settings including healthcare guidelines, policy research and educational development. This suggests that participant expectations of FCM were maintained following the method, and that the method yielded similar benefits to those in the literature.

During the pre-FCM interviews, participants spoke about their hopes that FCM could help to balance guideline requirements with the research evidence. In general healthcare guidelines represent a combination of committee member expertise and the systematic reviews of the evidence as presented by the technical team [1, 16]. However, where evidence is limited or low-quality, FCM can allow for the transparent and systematic recording of contributions from other sources, such as expert opinion.

Participants generally agreed that FCM required additional resources, although this presented differently for the technical team versus the committee. The technical team reported difficulties managing the increased responsibility and workload of generating the consensus statements, and the committee raised concerns about the credibility of the method and their confidence in using it. The generation of good quality statements is an important aspect to the FCM method, and better-quality statements are likely to increase the face validity and acceptability of the method for committee members. The statements used in the present study were in part generated from statements and recommendations from existing guidance, which was questioned by guideline members. It is likely that both the committee and the technical team would have benefited from more guidance and training to support the FCM process, alongside strong leadership from a FCM knowledgeable chair. Further research could identify whether resource intensiveness would reduce over time and experience of using FCM, or whether decision-making is more effective using FCM under specific circumstances.

Themes of FCM Credibility and Appropriate Application persisted across time-points. A frequently identified advantage of FCM was its transparent and robust approach of decision-making. Interviewees spoke of how this could help mitigate concerns from guideline consumers about the procedural validity of committee decision-making. Pre-FCM interviewees felt that greater clarity as to when to apply FCM would increase its credibility, and post-FCM interviewees elaborated when this could be. This included when committee expertise was low, for less cohesive groups, for low-quality evidence, or for when there is controversy about the evidence. Although guidance on the use of formal consensus methods exists within the NICE manual, [1] there was a lack of clarity about the criteria for its use which emerged in this study. Developing FCM best-practice could be supported by further developments in a FCM manual. This could delineate the criteria of application, guidelines about the additional resources required, and also allow for transparent evaluation of FCM.

The themes of “increased participation” and “discussion clarifying” were present across pre- and post- interviews and will be discussed together. Participants felt that FCM improved participation particularly when there were more dominant members of the group. Participants also spoke about using group discussion for clarification and noted that adequate time was required to balance free discussion with structured feedback prior to group voting and consensus.

Discussion has been found to increase expertise-sharing and to aid groups in evaluating the credibility of viewpoints [18]. Our findings demonstrate that OHPs such as pharmacists and dieticians appreciated discussion to gather feedback and develop ideas; however, doctors reported finding the discussions lengthy and frustrating. It might be that doctors are more practiced at making decisions under contexts of greater uncertainty, and thus find extended discussion unnecessary. Balancing unstructured with structured discussion might encourage the sharing of multiple perspectives, which has been suggested to support the quality, validity, and utility of guidelines [22]. Interdisciplinary dynamics within multidisciplinary teams (MDT) can reduce contributions from those who are perceived to have less power or expertise, for example consultants versus doctors, or social workers and nurses versus doctors; [23] although there is some evidence that this can be reduced through discussion taking place outside of work context and with the help of structured discussion [24]. Lay-members spoke about group processes the least, stating that they felt supported to participate through the use of designated service-user timeslots and separate discussions with the chair.

The benefits of anonymity for encouraging participation during the rating phase is an important feature of FCM, and this was discussed enough to reach threshold in the pre-FCM but not post-FCM interviews. FCM combines anonymous voting with free discussion to allow for dynamic feedback and collaboration from the group. The reduced presence of the theme of anonymity in the post-FCM could indicate that it was not a central feature of the FCM, perhaps because views became apparent during the subsequent discussion.

Group member continuity between meetings was identified as important for effective group decision-making by the pre-FCM interviews. Interviewees discussed how committee absences have a large impact on a guideline, particularly if the absent member had specific expertise for the area that was being discussed. However, it is important to acknowledge that group continuity is important within all committee meetings, it is not only relevant where FCM are being employed. The FCM procedure follows a clear recording of the creation and reiteration of statements, which could improve the communication of the decision-making process. This may help reduce repetition, increase focus for present committee members, and offer an incremental account of the decision-making to absent members. There was also a hope expressed that the transparent reporting of decision-making would increase the credibility of a guideline for absent stakeholders, but further investigation is warranted to explore potential benefits from their perspective.

A theme in the post-FCM interviews was the limited difference between FCM and informal consensus methods, which participants thought was due to high levels of agreement about the statements, particularly because the statements were derived from other guidance. Indeed, a separate theme in the post-FCM interviews was that the statements were ambiguous or repetitive even though they focused discussion. Interviewees thought a better use of FCM could be in areas when there is controversy or differences of view about the interpretation of evidence. This suggests an area for further development of the NICE manual for formal consensus to promote the effective use and practice-based evaluation of the FCM process.

During both pre- and post-FCM interviews, participants valued the discussion with other professionals and learning about a new method (pre-FCM interviews) and viewed this as a more complete and worthwhile process by using FCM (post-FCM interviews). This feedback suggests that FCM as a process is acceptable to participants despite the increased resources it was perceived to require. Overall, expectations of FCM provided in the pre-FCM interviews were similar to those experienced in the post-FCM interviews, the participants demonstrated a good understanding of the FCM process prior to the committee meeting, and afterwards views were generally consolidated.

### Strengths, limitations, and future directions

The qualitative data is context and sample specific, therefore the present findings should be generalised with caution. There are many themes that were specific to healthcare guideline development and the dynamics between the technical team and the committee members. The current study used a modified NGT method; however, the majority of the themes were consistent between the present study and previous research, which offers encouragement that the conclusions could be broadly relevant to NGT processes.

The present study was a preliminary investigation of FCM. The time, effort, and training required for the implementation of the FCM was as much a challenge for the technical team with no previous experience of FCM, as it was for committee members, and it was underestimated by the pilot project. There was also insufficient emphasis on grounding participants in manualised and clear criteria for the implementation of the FCM, which created difficulties for effective and systematic adoption of the method. An important reflection from this is that participants involved in FCM need more training and support than was offered.

The longitudinal design can follow the impact of the FCM exercise. However, differences must be interpreted with caution because the pre- and post-FCM time points used different interview questions, which could have influenced the themes. Although the conclusions drawn assume that differences in pre- and post-FCM themes occurred as a result of experiencing the FCM, there may have been other reasons for differences. These include participants increasing in confidence and familiarity in committee meetings and also with the researchers, which might have influenced their experience and openness in reporting. The second interviews took place up to three months after the FCM exercise, and therefore participant responses are also subjected to memory biases. The study did not follow-up participants who did not respond to the invitation for [25, 26] post-FCM interview. It could be that the post-FCM themes are biased by a self-selecting sample. Future research could focus on gathering views from all participants or using purposive sampling to mitigate against potential selection biases. Future research might also address some of the methodological concerns by triangulating interview data by using questionnaires or focus groups.

Researchers have previously described inconsistencies in reporting and implementation of FCM in research and practice, which creates a knowledge gap for appropriate application. The impact of this was also noted in the present study. Practice-based research and audit could help reduce the research-practice gap of FCM application to better inform it use.

In conclusion, FCM offers some advantages including increased participation, decision-making transparency, and widening the scope of sources that can be included as evidence statements. The effective application of FCM could benefit from including clear guidance on statement generation (where statements are sourced from and how they are framed to reflect uncertainty in the evidence), and which circumstances to apply FCM, (when there is controversy about the evidence, reduced committee expertise, less group cohesiveness, or limited or low-quality evidence), as it is important to only consider FCM when deemed an effective solution to decision making.

The present study highlighted the challenges involved in piloting and evaluating new methods of decision-making. Outside the laboratory FCM becomes contextualised in organisational dynamics, which could be further highlighted as the FCM process necessarily increases participation from a range of professionals with differing expectations and opinions. This might explain the complexities and variation of implementation experienced within this pilot study, which could benefit from better manuals and clear procedures for FCM guidelines.

## Conclusions

FCM is an acceptable alternative to informal consensus methods that has qualities specifically helpful to healthcare guidelines such as encouraging participation, inclusivity of a broad range of evidence, and managing group dynamics. More research is required to better understand when using formal consensus is most appropriate and effective.

## Electronic supplementary material

Below is the link to the electronic supplementary material.

Supplementary Material 1

Supplementary Material 2

## Data Availability

All data generated and/or analysed during the current study are not publicly available due to privacy reasons, but are available from the corresponding author on reasonable request.

## Abbreviations

FCM: Formal consensus method
MDT: Multidisciplinary team
NICE: National Institute for Health and Care Excellence
NGA: National Guideline Alliance
NGT: Nominal Group Technique
OHP: Other healthcare professionals
UCL: University College London
