# Supplementary material for: Committee experiences of using formal consensus in healthcare guidelines: a longitudinal qualitative study
Source: BMC Med Inform Decis Mak. 2023 Aug 2;23:147. doi: 10.1186/s12911-023-02220-5 (PMC10398942; doi:10.1186/s12911-023-02220-5)
Supplement: Supplementary file 1 — Supplementary Material 1 [file 12911_2023_2220_MOESM1_ESM.docx]

**Appendix 1.**

**Table 1. Participant demographics – those taking part in interviews pre formal consensus exercise**

| **Code** | **Age** | **Gender** | **Ethnicity** | **Profession** | **Prior Formal Consensus Experience** |
| --- | --- | --- | --- | --- | --- |
| A3 | 60s | Male | White British | Medical Consultant Doctor | None |
| A7 | 60s | Male | White British | Pharmacist | Previous experience once of Delphi method |
| A10 | 40s | Female | White British | Dietitian | Previous experience once of Delphi method |
| A13 | 30s | Female | White British | Service-user | None |
| A15 | 30s | Female | White Other | Guideline Lead | Previous multiple experiences of Delphi method |
| A18 | 30s | Female | White Other | Systematic Reviewer | None |
| B5 | 60s | Male | White British | Chair/GP  Medical Doctor | Previous experience once of Delphi method |
| B11 | 40s | Female | White British | Midwife | None |
| B16 | 40s | Female | White British | Service-User | None |
| B20 | 30s | Female | White British | Systematic Reviewer | None |
| B21 | 30s | Female | White Other | Systematic Reviewer | Previously used NGT once |
| B22 | 40s | Female | White Other | Health Economist | Previous multiple experiences of using NGT |

**Table 2. Participant demographics – those taking part in interviews post formal consensus exercise**

| **Code** | **Age** | **Gender** | **Ethnicity** | **Profession** | **Prior Formal Consensus Experience** |
| --- | --- | --- | --- | --- | --- |
| A7 | 60s | Male | White British | Pharmacist | Previous experience once of Delphi method |
| A10 | 40s | Female | White British | Dietitian | Previous experience once of Delphi method |
| A15 | 30s | Female | White Other | Guideline Lead | Previous multiple experiences of Delphi method |
| A18 | 30s | Female | White Other | Systematic Reviewer | None |
| B5 | 60s | Male | White British | Chair/GP  Medical Doctor | Previous experience once of Delphi method |
| B11 | 40s | Female | White British | Midwife | None |
| B20 | 30s | Female | White British | Systematic Reviewer | None |
| B21 | 30s | Female | White Other | Systematic Reviewer | Previously used NGT once |
| B22 | 40s | Female | White Other | Health Economist | Previous multiple experiences of using NGT |
