# Supplementary material for: Committee experiences of using formal consensus in healthcare guidelines: a longitudinal qualitative study
Source: BMC Med Inform Decis Mak. 2023 Aug 2;23:147. doi: 10.1186/s12911-023-02220-5 (PMC10398942; doi:10.1186/s12911-023-02220-5)
Supplement: Supplementary file 2 — Supplementary Material 2 [file 12911_2023_2220_MOESM2_ESM.docx]

**Appendix 2.**

**Pre-consensus interview questions**

1. What is your view of how decisions in this committee are made?
2. What is your experience so far of decision making when evidence is limited?
3. Do you think a more structured approach to decision making could be helpful, particularly when evidence is limited?
4. Can you tell me more about why you think that?
5. Are you familiar with formal and informal consensus methods?
   1. (if no) Offer definition: formal consensus methods are a structured approach to making decisions, and include Delphi, Nominal Group Technique, and RAND. A researcher will formulate evidence statements prior to the meeting of the committee about a topic based on pre-determined sources of evidence. These statements are then presented to each member of the committee who has an opportunity to rate them. These ratings are compiled and analysed for agreement. The statements that have the least agreement are revised based on feedback from the individuals. Individuals are then given the opportunity to re-rate the revised statements. Informal consensus methods offer no formal approach to the development of consensus statements once evidence is presented
   2. (if yes) can you tell me what your understanding is of formal consensus methods?
6. What do you think are the things that guide a committee to use a particular method, formal or informal?
   1. Anything else you can think of?
7. Do you have any experiences of using formal consensus methods?
8. If so, what are your own experiences of using formal consensus methods?
9. What do you think are the advantages and disadvantages of formal consensus methods?
10. What do you think is the impact of this (the disadvantage)?
11. What is your own view of formal consensus methods in guidelines in general?
12. How do you think formal consensus methods might be viewed by your colleagues?
13. Do you think use of formal consensus methods will affect how the guideline is broadly perceived?

**Post-consensus interview questions**

1. What was your experience overall of using the formal consensus Nominal Group Technique?
2. How well do you think the evidence statements were presented?
3. How representative were the statements of what you understand to be the key issues relevant to the guideline?
4. If at all, how did doing the NGT method shape group discussion?
5. How would you compare your experiences of using NGT and informal consensus methods?
6. Do you think it was an effective use of time?
7. How satisfied were you with the final statements that were generated?
8. Would you recommend the use of NGT as a decision making process?
   1. Why?
   2. When? (are there particular instances that you think formal consensus methods are more useful?)
9. Anything else?
